# Effects on P-Glycoprotein Expression after Blood-Brain Barrier Disruption Using Focused Ultrasound and Microbubbles

**DOI:** 10.1371/journal.pone.0166061

**Published:** 2017-01-03

**Authors:** Muna Aryal, Krisztina Fischer, Caroline Gentile, Salvatore Gitto, Yong-Zhi Zhang, Nathan McDannold

**Affiliations:** 1 Department of Radiology, Brigham & Women’s Hospital, Harvard Medical School, Boston, United States of America; 2 Department of Neurobiology, Harvard College, Cambridge, United States of America; 3 Department of Radiology, University of Pavia, Pavia, Italy; University of Kentucky Medical Center, UNITED STATES

## Abstract

Many blood-borne substances attempting to pass through the luminal membrane of brain endothelial cells are acted upon by a variety of metabolizing enzymes or are actively expelled back into the capillary lumen by embedded efflux transporters, such as Permeability-glycoprotein (Pgp). Overexpression of this protein has also been linked to multidrug resistance in cancer cells. Previous studies have shown that focused ultrasound (FUS), when combined with a microbubble agent, has ability to temporarily disrupt blood-brain barrier (BBBD). In this work, we investigated whether modulation of Pgp expression is part of the FUS-induced effects. We found that ultrasound can temporarily suppress Pgp expression. When BBBD was produced at 0.55 MPa, Pgp was suppressed up to 48 hours and restored by 72 hours. At 0.81 MPa, suppression can last 72 hours or longer. These findings support the idea that microbubble-enhanced FUS disrupts the functional components of the BBB through suppression of drug efflux.

## Introduction

P-glycoprotein (Pgp) is one of the proteins expressed naturally on the plasmatic membranes of endothelial cells at the blood-brain barrier (BBB). It protects the brain from harmful substances by excluding them from entering into the parenchyma from blood circulation. It is one of several so-called efflux pumps present at the BBB and in other organs. Overexpression of this protein excludes a wide range of therapeutics [1] for use as treatment for Central Nervous System (CNS) disorders. In the case of epilepsy and neurodegenerative disorders such as Amyotrophic Lateral Sclerosis (ALS), studies have suggested that Pgp expression may be elevated [2,3], potentially further restricting the delivery of drugs and resulting in less therapeutic benefits [4]. Additionally, with brain tumors, Pgp can be overexpressed in both the semi-permeable “blood-tumor barrier” (BTB) but also in the plasma membrane of tumor cells [5,6]. Overexpression of this protein and other efflux pumps are linked to multi-drug resistance against several anticancer drugs [7] and can result in tumors developing cross resistance to other therapeutics.

Different methods have been performed to inhibit Pgp expression and have shown promising outcomes in animal models, but clinical trials have proven to be unsuccessful in improving therapeutic efficacy [8]. Additionally, high doses seem to be required for complete inhibition, which can be life-threatening due to the lack of protection against harmful substances entering into the brain [9]. Currently, significant research effort is focused on identifying therapeutic targets within multiple signaling pathways that promote disease-related changes in Pgp activity [10] without inducing side effects. Having a technique that can selectively inhibit Pgp or other efflux pumps in targeted regions could be highly beneficial.

Ultrasound bursts, when combined with microbubbles, has emerged with great promise as a non-invasive and targeted method for drug delivery to the brain by temporarily disrupting the BBB [11]. This technique has several potential advantages over other approaches tested to overcome the BBB [12]. It is drug-neutral and enables delivery of a wide range of imaging agents and therapeutics such as antibodies, nanoparticles, and liposomally-encapsulated drugs to the brain [13–16] and enhances delivery to brain tumors [17–20]. Studies have also demonstrated that the BBB can be consistently disrupted without apparent neuronal damage [11,21–27], and it can be achieved using a clinical device [22].

The BBB is both a physical and functional barrier. Microbubble-enhanced focused ultrasound (FUS) has been shown to affect the tight junctions that restrict passive, paracellular diffusion into the brain as well as stimulating vesicular transcellular transport [28]. It is possible that it also could suppress drug efflux pumps such as Pgp. Indeed, others have shown in other contexts that ultrasound effects can suppress Pgp [29–33] but only limited studies have investigated this effect in CNS capillaries [34,35]. Here, we set out to characterize the possible interaction between the FUS exposures and Pgp expression in the BBB at different time points after sonication. We also examined Pgp expression after sonication at a higher level that produced vascular damage, and we examined whether acoustic emissions emitted by microbubbles during FUS-induced BBB disruption (FUS-BBBD) was correlated with the strength of Pgp expression at different time points after sonication.

## Materials and Methods

### Sonication system

An air-backed, single element, 690 kHz focused piezoelectric transducer (diameter/radius of curvature: 100/80 mm) generated the ultrasound field. It was driven by an arbitrary waveform generator (model 395, Wavetek) and RF amplifier (240L, ENI); electric power was measured with a power meter (E4419B, Agilent,) and dual-directional coupler (C5948-10, Werlatone). Reported exposure levels are absolute peak negative pressure amplitudes measured in water with a membrane hydrophone (Marconi; 0.5 mm diameter). Attenuation by the brain and rat skull is expected to reduce the pressure amplitude by ~30% at this frequency with additional uncertainty arising from standing waves within the skull and increases in skull thickness as the animal ages [36]. The pressure distribution of the transducer was mapped using a 0.2 mm needle hydrophone (Onda, Sunnyvale, CA); its half-maximum diameter and length were 2.3 and 12 mm, respectively. The transducer efficiency was measured using a radiation force-balance (Mettler Toledo, Columbus, OH).

Acoustic parameters were the same as in our previous study [18]. Five overlapping sonications consisting of 10 ms bursts delivered at a frequency 1 Hz for 60s were applied at a pressure amplitude of 0.55 or 0.81 MPa. Each sonication was combined with an intravenous injection of a microbubble-based ultrasound contrast agent (Definity; Lantheus) administered at the dose recommended for human ultrasound imaging (10 μl/kg). Each milliliter of Definity contains 1.2x10^10^ microbubbles that consist of perfluorocarbon gas-filled lipid shells with a mean diameter of 1.1–3.3μm. To facilitate the injections of such a small volume, the agent was diluted in PBS to 0.1 times its normal concentration. It was injected as a bolus approximately 9 s before each sonication, followed by a 0.2 ml saline flush.

### Experimental setup

**Fig 1:**
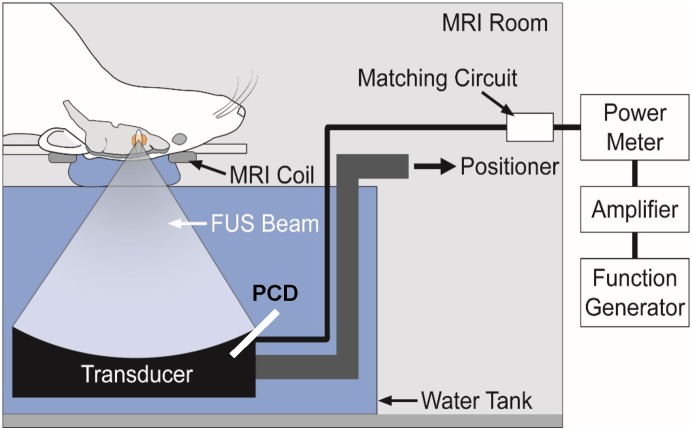
Schematic of the MRI-guided FUS system used in this work. The function generator, amplifier, and power meter were located outside the MRI room. A passive cavitation detector (PCD) was used to monitor the acoustic emissions.

The sonication system was operated within a clinical 3T MRI scanner (Signa; GE Healthcare). The transducer was immersed in a small tank of degassed, deionized water and attached to an MRI-compatible, manually-operated positioning system (Fig 1). The animal was laid supine on a tray above this tank, with a water bag providing an acoustic path to the dorsal surface of the head. Images were obtained with a 7.5 cm-diameter transmit/receive MRI surface coil. Before the rat experiments, we visualized heating in a silicone phantom using temperature-sensitive MRI to localize the acoustic focal point in the MRI space.

### Acoustic emissions monitoring system

The acoustic emissions were recorded using a piezoelectric passive cavitation detector (center frequency: 3.5 MHz, bandwidth: ±30%, diameter: 8 mm, Smart Materials, Sarasota, FL) transducer for every 10 ms burst. The procedure for measurement and analysis of the emissions are described in detail elsewhere [37]. Briefly, the passive cavitation detector was mounted next the transducer and aimed at the focal region to monitor the acoustic emission produced during sonication. The transducer was connected to a 0.4–1.7 MHz band-reject filter with a 20 dB gain (Model 3944, Krohn-Hite Corp, Brockton, MA, USA). The signals were recorded with a computer using a high-speed digitizing card (PXI-5124; National Instruments, Austin, Texas, USA). The time signal, frequency spectra, and magnitude of the emission at different harmonics were displayed in real-time during each sonication using software developed in-house in Matlab (Mathworks, Natick, MA, USA) and stored for later analysis. Emissions recorded during sonication with microbubbles was normalized to data obtained beforehand at the same locations and exposure levels without microbubbles. Frequency spectra were created from the time signals via fast Fourier transform. The strength of the relative integrated power spectra (in dB-Hz) were calculated over 20 Hz bins for the harmonics (FUS frequency × 2, 3, 4, etc.) and at sub- and ultra-harmonics (FUS frequency × 1/2, 3/2, 5/2, etc.). For each animal, the mean signal for all bursts and all sonications was calculated. Data in the range of the band-reject filter were excluded.

### Animals

All animal experiments were approved by the Institutional Animal Care and Use Committees of Harvard Medical School. Tests were performed in 31 Male Sprague-Dawley rats (Charles River Laboratories; ∼250 g). Animals were divided into two groups based on the pressure amplitude used for sonication, group A- 0.55 MPa (n = 13) and group B—0.81MPa (n = 15). Two animals from group A and one animal from group B were excluded because of errors that occurred during sonication or tissue sectioning. Before each procedure, the animals were anesthetized via intraperitoneal injections of ketamine (80 ml/kg/h) and xylazine (10 ml/kg/h). A catheter was placed in the tail vein, and the hair on the scalp was removed with clippers and depilatory cream. The body temperature was maintained throughout the experiment with a heated water pad.

### Magnetic resonance imaging

**Fig 2:**
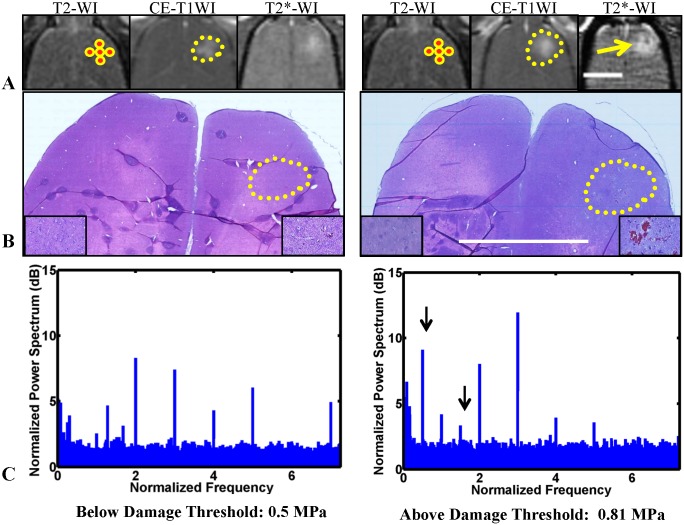
Sonication planning, monitoring, and assessment of FUS-induced BBBD after microbubble-enhanced sonication at 0.55 (left) and 0.81 (right) MPa. A. Axial MRI acquired during FUS-BBBD. T2-weighted imaging (T2WI) was used for treatment planning, contrast-enhanced T1-weighted imaging (CE-T1WI) to verify BBB permeabilization, and T2*-weighted imaging (T2*WI) to detect tissue damage. The targeted spots are indicated by circles; the yellow curves outline the extent of MRI contrast enhancement due to BBB permeabilization. Hypointense areas were evident in the focal plane at 0.81 MPa in T2*WI (arrow), indicating vessel damage and regions with extravasated erythrocytes; hyperintense areas in these images were due to MRI contrast agent extravasation. B. Appearance in H&E stained sections after BBBD at 0.55 MPa (left, 24 hours) and at 0.81 MPa (right, one hour). At 0.55 MPa, most of the sonicated region appeared unaffected, but in a few areas with tiny clusters of extravasated erythrocytes were seen (inset). Larger regions of erythrocytes were evident at 0.81 MPa. C. Examples of acoustic emissions recordings. These power spectra were normalized to recordings obtained without microbubbles. In each case, strong harmonic emissions were observed. Ultraharmonic and subharmonic emission were observed occasionally (arrows).

The experiments were performed under MRI guidance. T2-weighted imaging was used to plan the treatments (parameters: repetition time (TR): 2000 ms; echo time (TE): 79.7 ms; echo train length (ETL): 8; matrix size: 256×256; slice thickness/spacing: 1 mm/interleaved; field of view (FOV): 8 cm; number of excitations/averages (NEX): 2; bandwidth: 15.63 kHz). BBB disruption was evaluated using T1-weighted imaging (parameters: TR/TE: 500/13 ms; ETL: 4; matrix size: 256×256; slice thickness/spacing: 1 mm/interleaved; FOV: 8 cm; NEX: 4; flip angle: 90°; bandwidth: 15.63 kHz) after administration of the MRI contrast agent gadopentetate dimeglumine (Magnevist Gd-DTPA; Bayer Healthcare; 0.25 mL/kg). T2*-weighted imaging (parameters:TR/TE: 33.3/19 ms; matrix size: 256×256×28; slice thickness: 0.8 mm; FOV: 8 cm; NEX: 1; flip angle: 15°; bandwidth: 15.63 kHz) was used to examine whether petechiae, which are produced by excessive FUS exposures [11], did or did not occur (Fig 2A).

### Tissue preparation

All the animals were deeply anesthetized with ketamine/xylazine, and transcardially perfused with phosphate-buffered saline (PBS; 0.01 M phosphate-buffered 0.9% NaCl solution, pH 7.6) for ~10 sec, followed by perfusion with ~300 ml of 4% paraformaldehyde at 1 hour, 24 hours, 48 hours and 72 hours after FUS-induced BBBD. After fixation, the brains were removed and kept it in 4% paraformaldehyde overnight, cryoprotected in 30% sucrose at 4°C for 2–3 days, frozen, and sectioned with an 8 μm thickness using a cryostat (Mikron Instruments 82 Inc, Vista, CA).

### Immunohistochemistry

For potential unmasking of Pgp epitopes, the sections were pretreated with a mixture of 33% acetic acid and 66% ethanol for 10 minutes. Then the sections were washed in trisbuffered saline (TBS) and blocked for 60-min with 3% bovine serum albumin (BSA) and 11% normal rabbit serum (NRS) in TBS as a blocking solution. The primary antibody (monoclonal mouse anti-Pgp, C219, Dako) was diluted (15 μg/ml) in 1% BSA and 1% NRS. Sections were incubated overnight at 4°C. After washing in TBS, the sections were incubated with biotinylated rabbit anti-mouse antibody (Dako) in 1:200 for 60 min, followed by 90-min incubation with horseradish peroxidase–labeled streptavidin (1.65 μg/ml) at room temperature. 3, 3-Diamino-benzidine (DAB Peroxidase Substrate Kit, Vector Laboratories) was used for visualization. Histological analysis was performed on at least three stained sections of every brain.

### Scoring of Pgp-staining intensity in endothelial cells

**Fig 3:**
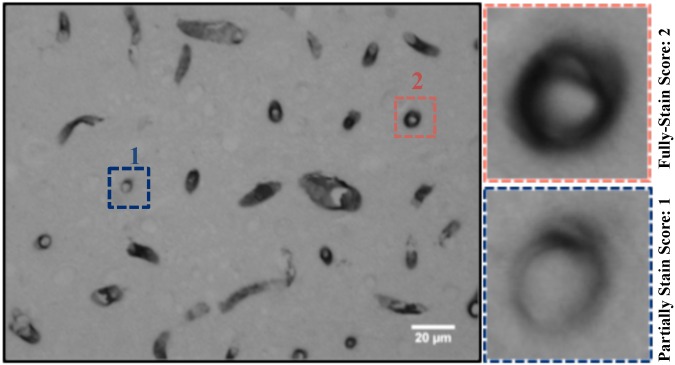
Method of scoring Pgp intensity after immunohistochemical staining. The microphotograph shows a section from the anterior right caudate putamen at a depth of 3 mm relative to the dural surface, the depth of the focal plane. The monoclonal antibody C219 was used for Pgp staining. Prominent immunoreactivity is evident in endothelial cells of capillaries. The intensity of the P-gp reaction was scored for each vessel in the section on a scale from 0–2, with 0 = not stained, 1 = partially-stained, 2 = fully-stained. Scale bar: 20 μm.

For analysis of the intensity of Pgp staining, a semiquantitative scoring system was used, similar to that used by others [38–40] for grading of endothelial and astrocyte staining. The intensity of Pgp expression was scored in nine 40× fields per region in the focal plane manually by an experienced investigator who took into account the background staining of the respective sections but was unaware to the FUS exposure parameters. The intensity of the Pgp reaction was scored on a scale from 0–2, with 0 = not stained, 1 = partially stained, 2 = fully stained. An example for values obtained by the grading scale is shown in Fig 3. In negative control sections for the staining background, produced with biotinylated secondary antibodies alone, background intensity was close to zero.

### Statistical analysis

The mean Pgp intensity score and the standard deviation were calculated for the sonicated region and in the same region in the non-sonicated hemispheres at one, 24, 48 and 72 hours after FUS-BBBD. The intensities among the sonicated and non-sonicated hemispheres were compared using one-tailed paired student's t-tests. For comparing the relative Pgp staining intensity and the harmonics strength between the two experimental groups, a one-tailed unpaired student’s t-test was used at each time point. Values of p < 0.05 were considered statistically significant.

## Results

### BBB permeabilization

BBB permeabilization and the presence or lack of petechiae were confirmed using contrast-enhanced and T2*-weighted MRI, respectively (Fig 2A). All sonicated regions demonstrated signal enhancement in contrast enhanced T1-weighted images after administration of Gd-DTPA, confirming BBB permeabilization induced by the sonications. In all animals in group B (pressure amplitude = 0.81MPa), one or more hypointense spots were observed in T2*–weighted MRI in the focal plane and on the brain surface in the cortex, suggesting the presence of significant petechiae. These dark spots were not observed in the focal plane in animals who received a lower ultrasound exposure in group A (pressure amplitude = 0.55MPa), but some spots were observed near the brain surface.

### Pgp expression at different time points

**Fig 4:**
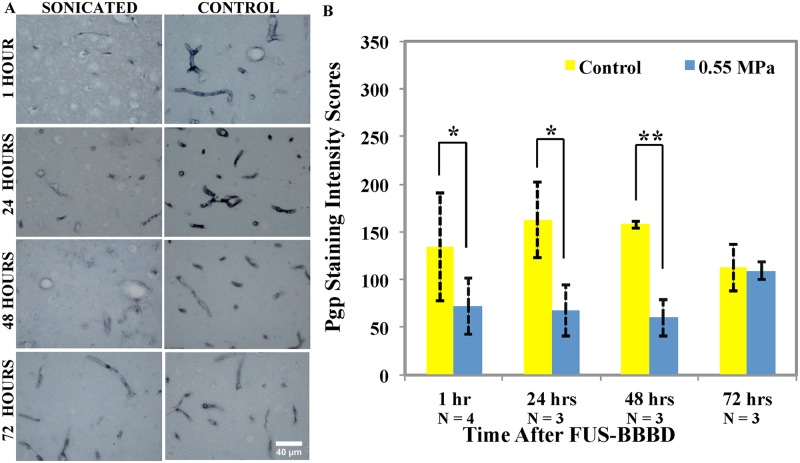
A. Representative microphotographs from regions in the sonicated and control hemispheres at different time points after BBBD induced by microbubble-enhanced sonication at 0.55MPa. Staining intensity was clearly reduced at one, 24, and 48 hours, but not at 72 hours. Fig 4. B. Pgp staining intensity scores at different time points after FUS-induced BBBD at 0.55MPa. A comparison of Pgp immunostaining in sonicated and control hemispheres revealed statistically significant differences in animals that were sacrificed one, 24 and 48 hours after BBBD (p<0.05, p<0.05, p<0.01, respectively), but not at 72 hours (p = 0.43). Scale bar: 40 μm.

Representative examples of microscopic images of Pgp staining in the sonicated and control hemispheres at different time points after FUS-induced BBBD at 0.55MPa (Group A) are shown in Fig 4A. In the control hemispheres, the mean Pgp staining intensities were 134.3 ± 56.4, 162 ± 39.5, 157.7 ± 4 and 113 ± 24.8 in the animals euthanized one (n = 4), 24 (n = 3), 48 (n = 3), and 72 hours (n = 3), after BBBD, respectively. In the sonicated hemisphere, the staining intensity was lower at one hour after FUS and recovered slowly over three days. Score of Pgp staining intensities were 72.3 ± 29.5, 68 ± 27, 60 ± 19 and 109.3 ± 9.3 in the sonicated hemisphere at one, 24, 48, and 72 hours after sonication, respectively. These values were 46%, 58%, 62% and 3% lower than the control hemispheres. The reductions at one, 24, and 48 hours were statistically significant (p < 0.05, p < 0.05 and p <0.01). Changes at 72 hours were not significant (p = 0.43) (Fig 4B).

**Fig 5:**
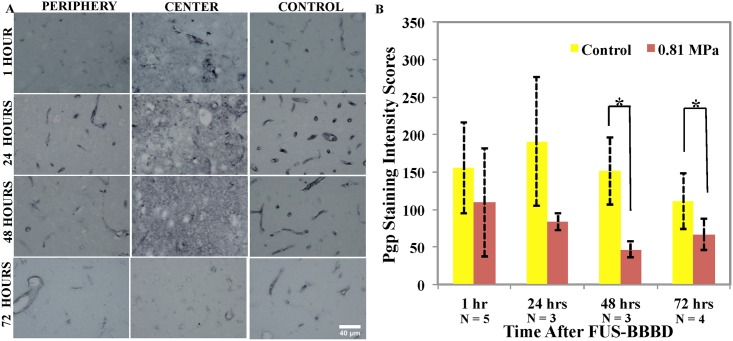
A. Representative microphotographs from regions in the sonicated and control hemispheres at different time points after BBBD induced by microbubble-enhanced sonication at 0.81 MPa. Except at 72 hours, the center of the sonicated region was diffusely stained. At the periphery, the staining intensity was reduced at all time points. Fig 5. B. Pgp staining intensity scores at different time points after BBBD induced by microbubble-enhanced sonication at 0.81MPa. A comparison of Pgp immunostaining in sonicated and control hemispheres found statistically significant differences between animals that were sacrificed 48, 72 hours after BBBD (p<0.05) but not at one (p = 0.25) or 24 hours (p = 0.094). Scale bar: 40 μm.

Representative examples of Pgp staining at different times after sonication at 0.81 MPa (Group B) are shown in (Fig 5A). In areas where vessel damage was evident (typically the center of the sonicated area), an increase in Pgp staining was evident throughout the brain parenchyma. This staining was not limited to endothelial or other discrete cells, but instead it was diffuse, particularly at one and 24 hours after sonication. In areas where vessel damage was not observed, a decrease in Pgp staining score was observed, typically at the periphery of the damaged region (Fig 5B). Mean Pgp staining intensity scores in these areas were 109.6 ± 72.8, 84 ±11.5, 46.7 ± 10.3 and 67 ± 21.5 in the sonicated hemisphere at one (n = 3), 24 (n = 3), 48 (n = 3), and 72 hours (n = 4) after BBBD respectively These values were lower than the control targets, where the mean scores were 155.2 ± 60.4, 191 ± 85.8, 151.3 ± 45.2 and 111.5 ± 37.3 at these times. The reductions (29%, 56%, 69% and 39% at one, 24, 48 and 72 hours, respectively) represented statistically significant differences at 48 and 72 hours (p<0.05), but not at one or 24 hours (p = 0.25, 0.094 respectively).

**Fig 6:**
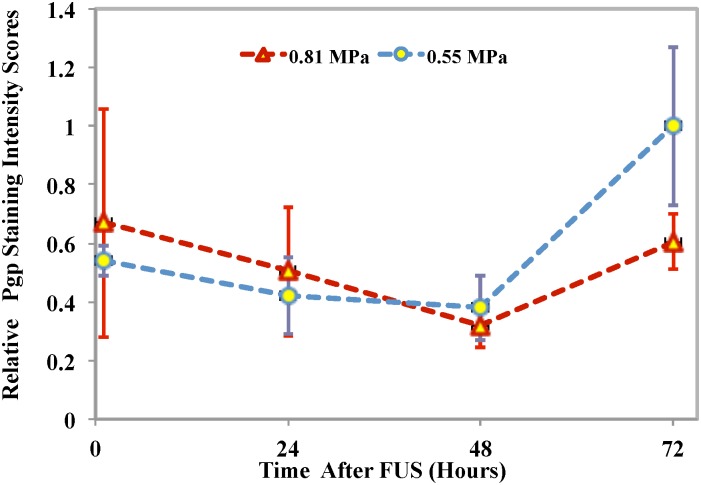
Relative Pgp staining intensity scores for the two exposure levels as a function of time. The scores were normalized to that measured in the control hemisphere. The difference in the relative intensity scores were not significant (p = 0.25, 0.3, 0.24 and 0.058, for one, 24, 48 and 72 hours, respectively).

Fig 6 shows compares of the relative Pgp staining intensity scores for the two different groups at different times after BBBD. Here, the scores were normalized to the measurements in the control hemisphere. The relative Pgp staining scores were 0.54 ± 0.053, 0.42 ±0.13, 0.38 ±0.11 and 1.0 ± 0.27 in Group A and 0.67 ± 0.39, 0.50 ± 0.22, 0.32 ± 0.075 and 0.61 ± 0.094 in group B. The intensity scores at one, 24, 48 and 72 hours were not significantly different between the two groups (p = 0.25, p = 0.3, p = 0.24 and p = 0.058 respectively). It should be noted that since the central portions of the sonicated region were diffusely stained after sonication 0.81 MPa, the intensity scores were made in the periphery where the pressure amplitude was presumably lower.

### Acoustic emission

**Fig 7:**
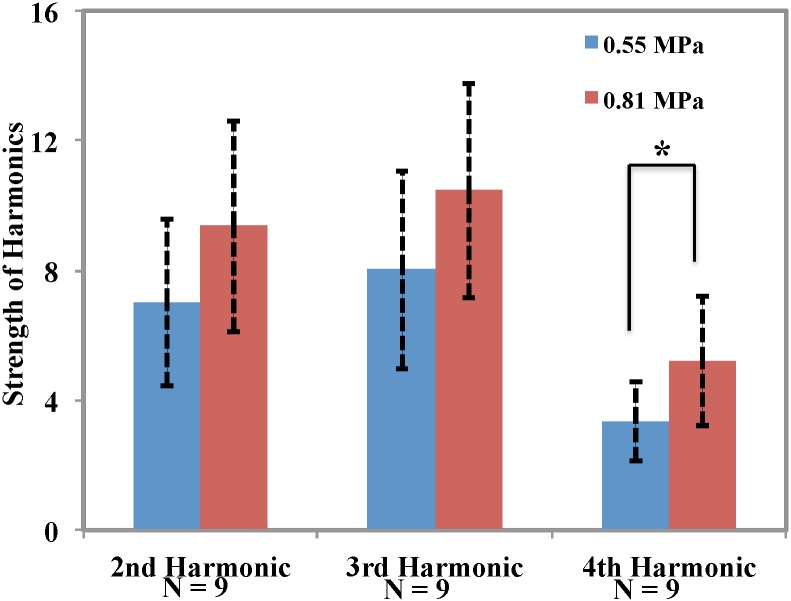
Strength of harmonics recorded during ultrasound mediated BBBD for the two exposure levels tested. The difference in the strength of the fourth harmonic was statistically significant (P<0.05), but not at the second or third harmonics (P = 0.054, 0.06, respectively).

Typical examples of acoustic emissions spectra of the two different groups are shown in Fig 2C. The data were obtained with a passive cavitation detector with a center frequency of 3.5 MHz and normalized to recordings acquired without microbubbles. After such normalization, only harmonic emission (FUS frequency × 2, 3, 4, etc.) and occasionally sub- or ultra-harmonic emission (FUS frequency × 1/2, 3/2, 5/2, etc.) were observed. The strength of the average emissions at the fourth harmonic (2.8 MHz, closest to the center frequency of the passive cavitation detector) were significantly higher (p<0.05) for sonications at 0.81 MPa than at 0.55 MPa (Fig 7), and a negative linear relationship with a good correlation (R^2^: 0.7) was observed between the fourth harmonic emissions strength and Pgp signal score in animals sacrificed at 48 hours. Such correlations were not observed for Pgp scores in animals sacrificed at other times. Differences in emissions strength at the other harmonics were not significant and no correlation was observed between these signals and Pgp staining score.

## Discussion

Pgp restricts the cellular uptake of drugs from blood circulation into the brain because it acts as an efflux pump at the BBB. Its expression is higher in certain disorders such as epilepsy and ALS. Because of its elevation in density, treatment of those diseases may be more challenging, even if promising therapeutics are identified [4]. Moreover, the presence of this protein in tumors contributes to cancer cells becoming resistant to chemotherapeutics and decreased treatment efficacy [10]. Although intense research efforts have been focused on the direct inhibition of Pgp, clinical trials testing Pgp inhibitors have largely proved to be unsuccessful in improving therapeutic efficacy, due perhaps to either incomplete suppression at the target or to side-effects caused by systemic inhibition [8].

Our results suggest that FUS and microbubbles can locally inhibit Pgp expression temporarily at the BBB in normal rats. They join other studies showing that such sonications modulate tight junctions and transcellular transport [41]. Together these studies suggest that the mechanical effects produced by the microbubbles on the vasculature suppress both the physical and functional components of the BBB. If these findings are translated to the clinic, they could enable localized Pgp inhibition without systemic side effects or the need to develop and test new Pgp inhibitor formulations.

Efflux pumps such as Pgp are involved in the process of clearing exogenous substances from the brain parenchyma. This clearance occurs along with clearance via the “glymphatic” system, where interstitial fluid is exchanged with cerebrospinal fluid flowing through paravascular channels [42], and potentially other factors [43,44]. Such clearance is different than what occurs in other tissues. Depending on the time it takes for other components of the BBB to be restored, it is possible that FUS-induced Pgp inhibition could lead to an enhancement in the time that drugs are present in the parenchyma. Such enhancement could increase drug efficacy. Thus, it would be interesting to compare BBB permeability and Pgp suppression at different times after sonication and to examine the clearance rate for substances that are and are not substrates for Pgp. It would also be interesting to investigate the effects of FUS-induced BBB disruption on the expression of Pgp and other efflux pumps on parenchymal cells in the brain and in brain tumor models.

At the lower exposure level, microbubbles-enhanced FUS succeeded in inhibiting Pgp expression up to 48 hours after BBBD; the expression recovered at 72 hours. At the higher exposure level, this expression had not fully recovered at 72 hours. Studies investigating the restoration of the barrier after sonication have found a half-life of a few hours [45–47]. Others have found low-level opening several days later [48]. Our findings are in agreement with a recent pharmacodynamics study of BBB opening by Sun et al [49], who found that with 4–5 μm size microbubbles, similar to size that we used in this study, that the barrier was disrupted three days after sonication, five at higher pressure amplitudes. When the BBB disruption was accompanied by vascular damage, the tissue appeared diffusely stained. This could have been due to cell membrane damage and the release of substances that stained positive for the anti-Pgp antibody. This diffuse staining restricted our scoring of affected blood vessels to peripheral regions and may explain why we did not see a significant decrease at one and 24 hours at the higher pressure amplitude. It also probably explains why the difference between the relative intensity scores were not significant between the two experimental groups at one and 24 hours in Fig 7 since the vessels were scored in the periphery of the sonicated area where the pressure amplitude was presumably lower.

The acoustic emissions produced during microbubble-enhanced sonication are a promising method to monitor and control FUS-induced BBB disruption [50] [37,51]. Prior works have demonstrated a correlation between the amount of tracers delivered after the sonications [37] and the volume of the disrupted area. Here, we observed a correlation between the harmonic emissions and the reduction in Pgp staining, but only for the animals sacrificed 48 hours after sonication, when the staining reduction was maximum. We expect that a lack of a correlation at earlier times was due to limitations of our detector and to the confounding effects produced in the central regions of the lesion where diffuse staining was observed. Future work is needed to verify these findings. Tests at different pressure amplitudes with more sensitive PCD may find additional information in the recorded cavitation activity that were missed here.

There are several other limitations to this study. The sample size was relatively small for the individual experimental groups, and more experiments could yield improved estimates of the length of time that Pgp is suppressed and reveal additional correlations between Pgp suppression and other factors. Use of different techniques to evaluate Pgp expression, such as reverse transcriptase polymerase chain reaction (RT-PCR) or RNA dot blot analysis [52], should be performed to confirm these findings.

Despite these limitations, these results are promising for the use of FUS to enhance drug delivery in the brain. Improvement of CNS drug delivery at the BBB through therapeutic manipulation of efflux transporter trafficking like Pgp ranks favorably as it has the potential to provide a noninvasive and targeted means to increase the delivery of a wide variety of drugs into the brain.

## Conclusions

This work suggests that FUS-induced BBB disruption can temporarily suppress Pgp expression in brain blood vessels. With sonication at 0.55 MPa, expression returned to baseline values at 72h. The suppression was longer with sonication at 0.81 MPa. The level of suppression was correlated with harmonic emission recorded with a passive cavitation detector, which may be useful for monitoring or controlling the level of induced suppression. These results support the idea that FUS and microbubbles disrupt drug efflux, part of the functional component of the BBB.
